# Chloroform in Intermittent Fever

**Published:** 1868

**Authors:** 


### CHLOROFORM IN INTERMITTENT FEVER.

Dr. D. Scott, of Bellefontaine, Iowa, has administered chloroform, in intermittent fever, as first recommended by Dr. E. McClellan, in the No. of this Journal for July, 1866,

(pp. 271–4) and April, 1867, (pp. 370–6) and he states (*Chicago Medical Journal,* Feb. 1866) that he has carefully noted its effect in upwards of fifty cases, with the following results: "In twenty cases, after the administration of one fluidrachm each, the chill was immediately arrested, with the exception of one case, in which the above dose was repeated in one hour; in eleven of the above cases, the febrile stage was probably abridged; of the remaining cases, the fever ran about as usual, all, with few exceptions, terminating in profuse perspiration; in eight of the cases, the paroxysm returned on the succeeding day, and nine on the second day, and three escaped, but were subsequently attacked in from seven to twenty days: of the remaining cases, no reliance was placed in the curative properties of the chloroform (which I only administered for the purpose of abridging the chill,) but was followed by large doses of sulph. quiniæ as soon as the sweating stage was established." In conclusion Dr. S. says that chloroform is a valuable and safe hypnotic in the dose of one fluidrachm, in the cold stage of intermittent fever, and never fails to arrest the chill, the patient falling into a refreshing slumber as described by Dr. Dr. McClellan. Dr. S. administered it, like Dr. McClellan, undiluted.—*American Journal Medical Science.*

